# Ferroptosis and Sterol Biosynthesis Dysregulation in Granulosa Cells of Patients with Diminished Ovarian Reserve

**DOI:** 10.3390/antiox14060749

**Published:** 2025-06-17

**Authors:** Yang Yu, Yali Shan, Jiani Lu, Yexing Xian, Zhengshan Tang, Xinyu Guo, Yan Huang, Xin Ni

**Affiliations:** 1 Department of Gynecology and Obstetrics, Xiangya Hospital Central South University, Changsha 410000, China; fishsheep0622@163.com (Y.Y.); 13786380104@163.com (Y.S.); 2 International Collaborative Research Center for Medical Metabolomics, Xiangya Hospital Central South University, Changsha 410000, China; tangzhengshan1992@163.com; 3 Department of Rehabilitation, Shanghai General Hospital, Shanghai Jiao Tong University School of Medicine, Shanghai 200025, China; lvjiani0713@163.com; 4 Center of Reproductive Medicine, General Hospital of Southern Theatre Command, Guangzhou 510010, China; xianstar2007@163.com (Y.X.); iris92@126.com (X.G.); 5 National Clinical Research Center for Geriatric Disorders, Xiangya Hospital Central South University, Changsha 410000, China

**Keywords:** diminished ovarian reserve, granulosa cells, ferroptosis, cholesterol synthesis, steroid hormone synthesis, aromatase

## Abstract

Granulosa cell (GC) dysfunction contributes to diminished ovarian reserve (DOR). We collected GC and follicular fluid samples from the patients of normal ovarian reserve (NOR) and DOR. RNA-seq of GCs showed that cholesterol/sterol metabolism and biosynthesis and extracellular matrix organization were enriched in the DOR group. Metabolomics of follicular fluid revealed enrichment in steroid hormone biosynthesis, tryptophan metabolism, and fatty acid β-oxidation in DOR. The apoptosis rate was increased, whereas the proliferative rate was decreased in GCs of DOR. The Prussian blue staining rate was increased whilst GPX4 and SLC7A11 expression were downregulated in GCs of DOR. Mitochondrial morphology displayed the features of ferroptosis in GCs of DOR. FSHR, CYP19A1, NR5A1, and phosphorylated CREB levels were substantially downregulated in GCs, accompanied by increased androgen levels in follicular fluids in DOR. The key factors linked to the mevalonate pathway, HMGCR, SQLE, and SREBF2, were robustly increased in DOR. FSHR and NR5A1 levels were correlated with CYP19A1 levels, whilst CYP19A1 levels were positively correlated with GPX4 and SLC7A11 levels. Our findings indicate ferroptosis and dysregulation of cholesterol/sterol metabolism and biosynthesis occurrence in GCs of DOR, which might be associated with reduced FSHR signaling and decreased conversion of androgen to estrogen.

## 1. Introduction

Diminished ovarian reserve (DOR) is a condition characterized by a decline in ovarian function, marked by a reduction in the quantity, quality, and developmental potential of oocytes within the ovaries [1]. This condition contributes to ovulation disorders and compromised oocyte quality, ultimately impairing female fertility. The prevalence of DOR is increasing, affecting approximately 10% of young women [2]. So far, no clinical management approach has been able to blunt this increase in the incidence of DOR in the past 10 years [1,3]. Such failure is largely due to the limited understanding of the etiology and pathogenesis underlying DOR in the majority of cases.

Granulosa cells (GCs) are highly specialized somatic cells that form multi-layered structures surrounding the oocyte. They play a pivotal role in folliculogenesis, facilitating the transition of follicles from the primordial to the pre-ovulatory stage by supporting follicular growth and development through paracrine signaling pathways [4,5]. The quality and quantity of GCs are crucial for the maturation of the oocyte and early embryo development competence [6,7]. In clinical practice, GCs can be obtained from patients undergoing assisted reproductive technology (ART), providing readily accessible biological samples for studying female follicle development and offering an ethical approach that avoids the unnecessary use of female oocytes. GCs are therefore regarded as one of the most effective noninvasive methods for investigating ovarian function. Thus, a comprehensive understanding of molecular pathways within granulosa cells in DOR patients can help to elucidate the pathogenesis of DOR. Some studies have identified several signaling pathway changes in GCs from DOR patients using transcriptomic and metabolomic approaches [8,9,10,11,12,13,14,15]. However, the findings are not consistent across studies due to variations in study populations, differences in sample sizes, and insufficient validation at both mRNA and protein levels.

The aims of the present study were to investigate potential molecular mechanisms underlying the pathogenesis of DOR by exploring key pathways associated with cellular abnormalities in GCs. To achieve the goals, we recruited 212 patients and analyzed transcriptome profiles in a total of 116 samples of mural GCs obtained from DOR patients and the controls of normal ovarian reserve (NOR). Subsequently, we employed quantitative real-time RT-PCR (qPCR), immunofluorescence, electron microscopy, and metabolomic profiling of follicular fluid to identify the key molecular pathways. Our results revealed that GC dysfunction and cellular injury were associated with the occurrence of ferroptosis, as well as reprogramming of sterol and cholesterol synthesis and metabolism in GCs. These abnormalities may be attributed to reduced FSHR expression and conversion of androgens to estrogens in GCs. Our findings provide critical insights into potential therapeutic targets for protecting or restoring ovarian reserve.

## 2. Materials and Methods

### 2.1. Patients and Sample Collection

This study was approved by the Ethics Committee of the General Hospital of the Southern Theatre Command (Approval No. NZLLKZ2024052). Informed consent was obtained from all subjects.

The patients with DOR and NOR who underwent controlled ovarian stimulation for in vitro fertilization (IVF) or intracytoplasmic sperm injection (ICSI) at the Centre for Reproductive Medicine, General Hospital of Southern Theatre Command, were recruited from February 2021 to September 2024. The criteria for DOR included assessments of AMH, basal FSH levels, and bilateral antral follicle counts (AFCs) [16]. Patients were classified as DOR if at least two of the following three criteria were met, i.e, bilateral AFC ≤ 7, AMH ≤ 1.1 ng/mL, or a menstrual basal FSH level between 10 and 25 mIU/mL. The NOR group comprised the women who underwent IVF treatment due to male factors, tubal factors, or endometrial factors, and exhibited serum anti-Müllerian hormone (AMH) levels ≥ 1.5 ng/mL, bilateral AFCs ranging from 8 to 24, and basal follicle-stimulating hormone (FSH) levels < 10 IU/L. Bilateral AFCs on menstrual cycle days 2–3 were calculated by transvaginal ultrasonography. Patients with a history of radiotherapy, ovarian surgery, polycystic ovary syndrome, polycystic ovaries, or endometriosis were excluded from this study. All patients enrolled underwent a standardized gonadotropin-releasing hormone (GnRH) antagonist protocol for controlled ovarian stimulation as described previously [7,17]. Briefly, the women received recombinant or urinary gonadotropins on day 2 or day 3 of the menstrual cycle, and GnRH antagonists were administered according to follicular diameter. When one or more follicles reached diameters of up to 20 mm, an injection of human chorionic gonadotropin (HCG) was administered to induce follicular maturation. Mature follicles were received via ultrasound-guided puncture 34–36 h later. After the cumulus–oocyte complexes were transferred into IVF culture media, the remaining follicular fluid was centrifuged at 500× *g* for 5 min, and then the supernatants were collected, snap-frozen in liquid nitrogen, and stored at −80 °C. The cell aggregates were carefully added to the upper layer of 50% Percoll (Solarbio, Beijing, China) and centrifugation at 500× *g* for 15 min. The granulosa cell layer was aspirated, resuspended in 5 mL PBS, and centrifuged again at 500× *g* for 5 min. Finally, the granulosa cells were either snap-frozen in liquid nitrogen and stored at −80 °C or fixed in 4% formalin for further analysis. The purity of the collected GCs was assessed by using immunofluorescence of GC marker FSHR, and the results showed that more than 97% of cells were FSHR positive (Supplemental Figure S1).

### 2.2. Measurement of Hormones in Blood

The levels of FSH, luteinizing hormone (LH), estradiol (E2), progesterone (P4), testosterone (T), AMH, and FSH in the serum of patients were determined by commercial radio-immunoassay kits at the clinical lab in the General Hospital of the Southern Theater Command. Basal FSH, E2, LH, and P4 levels were detected on the menstrual cycle days 2–3. In addition, the levels of FSH, E2, LH, and P4 were also detected on the HCG trigger day.

### 2.3. Transcriptome Analysis

A total of 116 samples (62 DOR and 54 NOR) were used for RNA-sequencing. The analysis was performed by Novogene Co., Ltd. (Beijing, China). Briefly, FPKM of each gene was calculated based on the length of the gene and the read count mapped to this gene. Subsequently, differential expression analysis of the two groups was performed using the DESeq2 R package (1.20.0). DESeq2 employs statistical methods based on a negative binomial distribution model to assess differential expression in digital transcriptome profiling data; the Benjamini–Hochberg procedure was applied to control false discovery rates through *p*-value adjustment. Transcripts meeting the dual criteria of adjusted *p*-value (padj) < 0.05 accompanied by an absolute fold change > 2 were identified as differentially expressed genes (DEGs). Kyoto Encyclopedia of Genes and Genomes (KEGG) and Gene Ontology (GO) enrichment analysis was conducted using the Metascape website (https://metascape.org/gp/index.html, Accessed on 15 May 2024). Gene Set Enrichment Analysis (GSEA) was performed by using GSEA v4.3.2 software (http://software.broadinstitute.org/gsea/index.jsp, Accessed on 15 May 2024). DEG interactions were analyzed using the online tool STRING (https://string-db.org/, Accessed on 15 May 2024). The interactions with interaction scores higher than 0.4 were selected for protein–protein interaction (PPI) network construction. Cytoscape software (https://www.cytoscape.org/, Cytoscape_v3.10.3, Accessed on 15 May 2024) was used for network visualization and further analysis. Key modules of the PPI network were identified using the MCODE plugin. Hub genes of the network were identified using the cytoHubba plugin. Transcription factors of the DEGs were predicted using the online tool ChEA3 (https://maayanlab.cloud/chea3/, Accessed on 15 May 2024).

### 2.4. Quasi-Targeted Metabolomics

Metabolomic analysis was conducted on 41 follicle fluid samples (25 NOR and 16 DOR) by Novogene Co., Ltd. (Beijing, China). The samples were extracted using prechilled 80% methanol buffer, and the supernatants were collected for LC-MS/MS analysis. The LC-MS/MS analyses were carried out using an ExionLC™ AD system (SCIEX) coupled with a QTRAP^®^ 6500+ mass spectrometer (SCIEX). Chromatographic separation was performed on an Xselect HSS T3 column (2.1 × 150 mm, 2.5 μm) using a 20 min linear gradient at 0.4 mL/min flow rate under dual polarity conditions with mobile phases consisting of 0.1% formic acid in water (eluent A) and 0.1% formic acid in acetonitrile (eluent B), where the QTRAP^®^ 6500+ mass spectrometer operated with curtain gas of 35 psi, medium collision gas, an ion source temperature of 550 °C, gas 1/gas 2 both at 60 arbitrary units, and employing polarity-specific IonSpray voltages (5500 V for positive mode and −4500 V for negative mode), while metabolite detection utilized multiple reaction monitoring (MRM) transitions from the Novogene in-house database with Q3-based quantification, processing raw LC-MS/MS data in SCIEX OS v1.4 using defined parameters (minimum peak height: 500 counts; signal-to-noise ratio ≥ 5; gaussian smoothing width 1 point) to generate normalized peak areas reflecting relative metabolite abundance, followed by metabolite annotation through KEGG (http://www.genome.jp/kegg/, Accessed on 15 May 2024), HMDB (http://www.hmdb.ca/, Accessed on 15 May 2024), and LipidMaps databases (http://www.lipidmaps.org/, Accessed on 15 May 2024), where differential metabolites were identified using dual thresholds (*p*-value < 0.05 with fold change > 1.2 or <0.83) visualized via ggplot2-generated volcano plots (−log10(*p*-value) vs. log2(FC)), ultimately conducting metabolite set enrichment analysis through MetaboAnalyst 6.0 (https://www.metaboanalyst.ca/, Accessed on 15 May 2024) using over-representation analysis (ORA) and quantitative enrichment analysis (QEA) methods with KEGG/SMPDB (https://smpdb.ca/, Accessed on 15 May 2024) pathway references.

### 2.5. Q-PCR

Q-PCR was performed on 61 samples (34 NOR and 27 DOR) to validate mRNA expression levels. Following RNA extraction from granulosa cells using TRIzol^®^ Reagent (Takara Bio, Kyoto, Japan), 2 μg total RNA was reverse-transcribed with SuperScript™ IV Reverse Transcriptase (Invitrogen, Carlsbad, CA, USA) to synthesize cDNA. Gene-specific primers (Supplementary Table S1, Tsingke Biotechnology Co., Ltd., Beijing, China) were employed in amplifications executed on a MiniOpticon™ Real-Time PCR System (Bio-Rad Laboratories, Hercules, CA, USA) with the following reaction parameters: 20 μL reaction volumes containing 2.0 μL cDNA template, 0.2 μM forward/reverse primers, and ChamQ Universal SYBR qPCR Master Mix (Vazyme Biotech Co., Ltd., Nanjing, China). Normalization was performed against the endogenous control β-actin, with reaction specificity verified through melt curve analysis. Relative quantification of target gene expression was calculated using the comparative Ct (threshold cycle) method with the arithmetic formula (2 − ΔΔCt).

### 2.6. Immunofluorescence (IF)

GCs from 21 patients (12 NOR and 9 DOR) were fixed in 10% buffered formalin overnight and then embedded in paraffin. Paraffin sections (5 μm) were rehydrated and microwaved in citric acid buffer to retrieve antigens, and then incubated with 10% BSA for 1 h. Immunofluorescence staining was performed through sequential incubations with species-specific primary antibodies (Supplementary Table S2) at 4 °C for 16 h, followed by three PBS washes (5 min/wash) and subsequent 2 h incubation with Alexa-Fluor-conjugated secondary antibodies (1:1000; species-matched IgG) under light-protected conditions at 25 °C. Nuclear counterstaining was achieved using DAPI (0.1 μg/mL, 5 min), with specimen visualization conducted on an Olympus fluorescence microscope (Tokyo, Japan). To ensure analytical objectivity, all imaging procedures and quantitative assessments were executed by investigators blinded to sample group assignments.

### 2.7. TUNEL Assay

The TUNEL staining was conducted by using a commercial kit (TUNEL FITC Apoptosis Detection Kit, Vazyme Nanjin, China). Briefly, a 5 μm paraffin-embedded section of GCs was deparaffinized, rehydrated, and then incubated at 25 °C for 15 min in a diluted proteinase K solution, followed by a rinse with PBS. The sections were subsequently stained with the TUNEL reaction mixture (C1090, Beyotime, Shanghai, China) and incubated for 2 h at 37 °C in the dark. Fluorescence was measured using an Olympus fluorescence microscope (Tokyo, Japan).

### 2.8. Transmission Electron Microscopy (TEM)

The fresh GCs from 14 patients (7 NOR and 7 DOR) were fixed in the 2.5% glutaraldehyde solution for 24 h. Then, the cells were postfixed with 1% osmium tetroxide (0.1 M phosphate buffer, pH 7.4) for 2 hr at 4 °C, followed by sequential ethanol dehydration (50%, 70%, 90%, and 100% × 3), and embedded in epoxyresin. Ultrathin sections (50 nm nominal thickness) prepared using an ultramicrotome were double-contrasted with uranyl acetate (5% in 50% ethanol, 15 min) and lead citrate (Reynolds’ formulation, 5 min), and observed with a Hitachi HT7700 (Hitachi, Tokyo, Japan).

### 2.9. Prussian Blue Staining

Prussian blue staining was conducted by using a commercial kit (Wuhan Servicebio Technology Company, Wuhan, China). Briefly, a 5 μm paraffin-embedded section of GCs from 38 patients (22 NOR and 16 DOR) was deparaffinized and rehydrated. The slides were then immersed in Prussian blue staining solution for 1 h, followed by two washes with distilled water. Subsequently, the slides were counterstained with hematoxylin solution. The stained slides were observed under an Olympus microscope (Tokyo, Japan).

### 2.10. Statistical Analysis

Statistical analyses were conducted using SPSS version 21. Data are presented as mean ± standard error of the mean (SEM). Normality of distribution was evaluated with the Shapiro–Wilk test. For comparisons between two groups, a two-tailed Student’s *t*-test was employed on the data with a normal distribution. Pearson analysis was used to determine correlations between variables. A *p*-value of less than 0.05 was considered indicative of statistical significance.

## 3. Results

### 3.1. Baseline Information of the Participants

The characteristics of the patients recruited in this study are listed in Table 1. As expected, the average AFCs were significantly reduced in the DOR group compared with the NOR group. Circulatory baseline FSH level was higher, whereas the baseline AMH level was lower in the DOR group compared to the NOR group. Of note, the baseline LH level was lower, and the T level was also lower in DOR patients than in NOR patients. However, circulatory levels of FSH and LH on the day of HCG stimulation were higher in the DOR group than those in the NOR group. In contrast, circulatory E2 and P4 levels on the day of HCG were lower in the DOR group than those in the NOR group.

### 3.2. Many Pathways, Including Steroid Synthesis, Cholesterol Metabolism, and Extracellular Matrix, Are Dramatically Changed in DOR Patients

**Figure 1:**
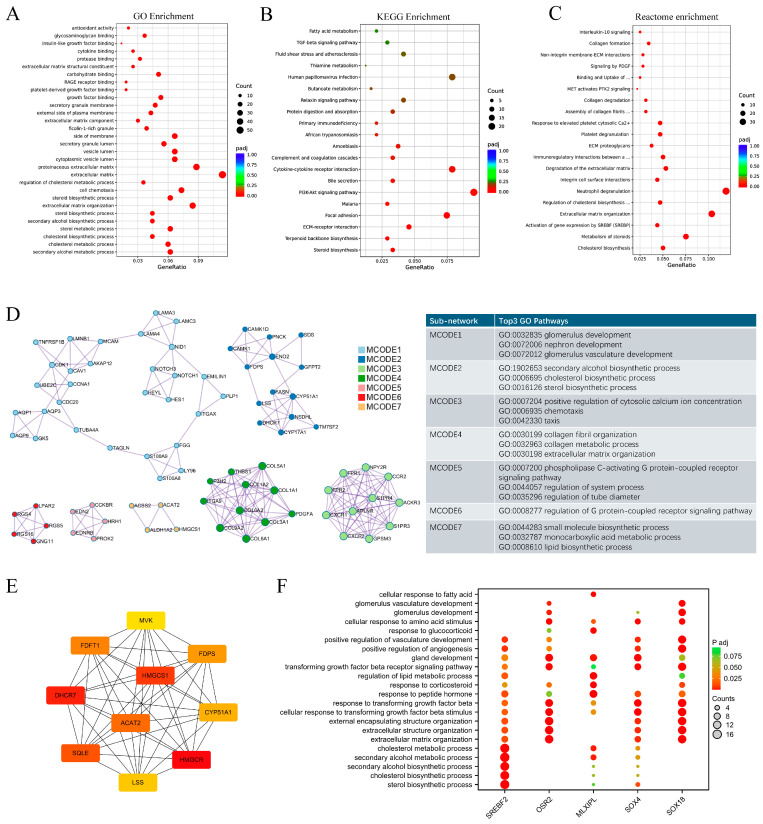
**Overview of transcriptomic changes in human granulosa cells from NOR and DOR patients.** RNA-seq was conducted on 116 GC samples (62 DOR and 54 NOR). (**A**–**C**) GO, KEGG, and Reactome pathway enrichment analysis of DEGs in DOR vs. NOR. (**D**) MCODE analysis of PPI network of DEGs in DOR vs. NOR. (**E**) Top 10 hub genes of PPI network of DEGs in DOR vs. NOR. (**F**) Predicted TFs of DEGs in DOR vs. NOR and top five GO pathways enriched in targeted genes of each predicted TF.

Transcriptome analysis showed that 583 genes were altered in the DOR group compared to the NOR group, among which 153 genes were upregulated, whilst 430 genes were downregulated (Supplemental Figure S2A). Antioxidant activity, the external side of the plasma membrane, cholesterol metabolic process, cholesterol biosynthetic process, sterol metabolic process, sterol biosynthetic process, extracellular matrix organization, etc., were enriched in GO enrichment analysis (Figure 1A). Steroid biosynthesis, ECM–receptor interaction, focal adhesion, etc., were enriched in KEGG enrichment (Figure 1B). Cholesterol biosynthesis, metabolism of steroids, activation of gene expression by SREBP, and degradation of extracellular matrix were displayed in Reactome enrichment analysis (Figure 1C).

Seven key sub-networks were detected from the PPI network of differentially expressed genes (DEGs) in DOR vs. NOR using MCODE analysis (Figure 1D). Based on the GO pathway analysis, these seven modules are associated with glomerulus development, cholesterol biosynthetic process, sterol biosynthetic process, collagen metabolic process, extracellular matrix organization, phospholipase C-activating G protein-coupled receptor signaling pathway, regulation of G protein-coupled receptor signaling pathway, lipid biosynthetic process, etc. Figure 1E shows the hub genes, which include HMGCR, HMGCS1, SQLE, DHCR7, CYP51A1, and FDPS, which are the genes in the cholesterol synthesis pathways. Potential transcriptional factors (TFs) of the DEGs were predicted using ChEA3. Figure 1F shows the potential TFs and the GO pathways of their target genes.

### 3.3. Common and Differential Pathways Among DOR Patients Under and over 35 Years Old (Y/O)

Next, we further analyzed DEGs between NOR and DOR in different age groups, i.e., <35 Y/O groups and ≥35 Y/O groups.

**Figure 2:**
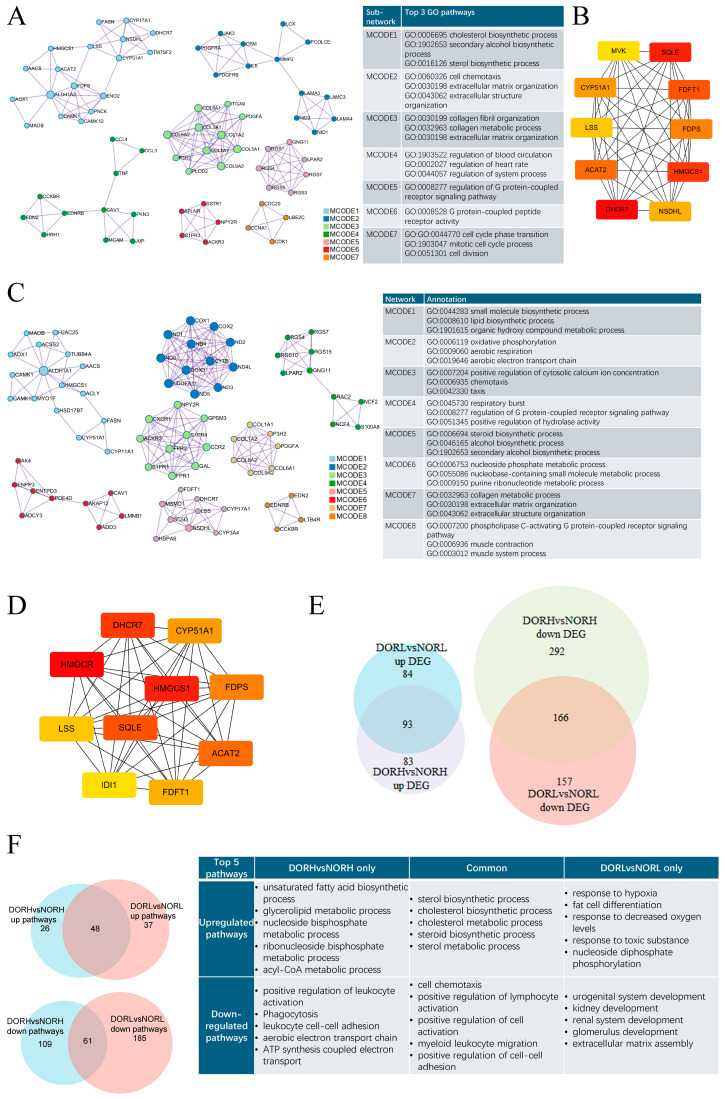
**Common and differential pathways among women under and over 35 years old.** (**A**) MCODE analysis of PPI network of DEGs in DORL vs. NORL. (**B**) Top 10 hub genes of PPI network of DEGs in DORL vs. NORL. (**C**) MCODE analysis of PPI network of DEGs in DORH vs. NORH. (**D**) Top 10 hub genes of PPI network of DEGs in DORH vs. NORH. (**E**) Venn diagram of DEGs in DORL vs. NORL and DORH vs. NORH. (**F**) Venn diagram of GO pathways based on analysis of DEGs in DORL vs. NORL and DORH vs. NORH. DORH, the DOR patients over 35 years old. NORH, the NOR patients over 35 years old. DORL, the DOR patients under 35 years old. NORL, the NOR patients under 35 years old.

It was found that 177 genes were upregulated whilst 324 genes were downregulated in DOR patients under 35 years old compared to the age-matched healthy group (Supplemental Figure S3A). Supplemental Figure S3B showed GO enrichment analysis including antioxidant activity, side of membrane, cholesterol metabolic process, cholesterol biosynthetic process, sterol metabolic process, sterol biosynthetic process, steroid biosynthetic process, extracellular matrix, growth factor binding, etc. Supplemental Figure S3C shows KEGG enrichment analysis including ECM–receptor interaction, steroid biosynthesis, the PI3K-Akt signaling pathway, butanoate metabolism, thiamine metabolism, etc. MCODE analysis of the PPI network identified seven key modules that are related to the cholesterol biosynthetic process, extracellular matrix organization, regulation of blood circulation, regulation of the G protein-coupled receptor signaling pathway, G protein-coupled peptide receptor activity, and cell division (Figure 2A). The hub genes were the genes linked to cholesterol biosynthesis (Figure 2B).

In ≥35 Y/O groups, 177 genes were upregulated whilst 458 genes were downregulated in DOR patients (Supplemental Figure S3D). GO enrichment demonstrated the enriched pathways, including NADH dehydrogenase activity, cofactor binding, the mitochondrial respiratory chain, the cholesterol biosynthetic process, the sterol metabolic process, the sterol biosynthetic process, the steroid biosynthetic process, regulation of the cholesterol metabolic process, regulation of the steroid biosynthetic process, the extracellular matrix, etc. (Supplemental Figure S3E). KEGG enrichment showed steroid biosynthesis, hematopoietic cell lineage, ovarian steroidogenesis, cortisol synthesis and secretion, protein digestion and absorption, primary immunodeficiency, cytokine–cytokine receptor interaction, aldosterone synthesis and secretion, cell adhesion molecules, etc. (Supplemental Figure S3F). Nine key modules were identified via MCODE analysis (Figure 2C). They are associated with the lipid biosynthetic process, oxidative phosphorylation, the positive regulation of cytosolic calcium ion concentration, the regulation of the G protein-coupled receptor signaling pathways, the nucleoside phosphate metabolic process, extracellular matrix organization, the phospholipase C-activating G protein-coupled receptor signaling pathway, etc. Interestingly, hub genes were also the genes linked to cholesterol synthesis (Figure 2D).

Nineteen upregulated genes and 170 downregulated genes were found between DOR ≥ 35 years old and DOR < 35 years old. There were 259 common DEGs and 616 differential DEGs between DOR vs. NOR under 35 Y/O and DOR vs. NOR over 35 Y/O (Figure 2E). Enrichment analysis showed the common upregulated pathways, including the sterol biosynthetic process, the cholesterol biosynthetic process, the cholesterol metabolic process, the steroid biosynthetic process, the sterol metabolic process, and downregulated pathways, including cell chemotaxis, positive regulation of cell–cell adhesion, positive regulation of lymphocyte activation, and positive regulation of cell activation in KEGG enrichment (Figure 2F).

In <35 groups, upregulation pathways in DOR vs. NOR were responses to hypoxia, fat cell differentiation, nucleoside diphosphate phosphorylation, etc. Downregulation pathways in DOR vs. NOR were urogenital system development, kidney development, extracellular matrix assembly, etc. In over 35 Y/O groups, upregulation pathways in DOR vs. NOR were the unsaturation fatty acid biosynthetic process, the glycerolipid metabolic process, the acyl-CoA metabolic process, etc., whilst downregulation pathways in DOR vs. NOR were only positive regulation of leukocyte activation, phagocytosis, aerobic electron transport chain, etc.

### 3.4. Metabolome Profile in Follicle Fluid of NOR and DOR Groups

**Figure 3:**
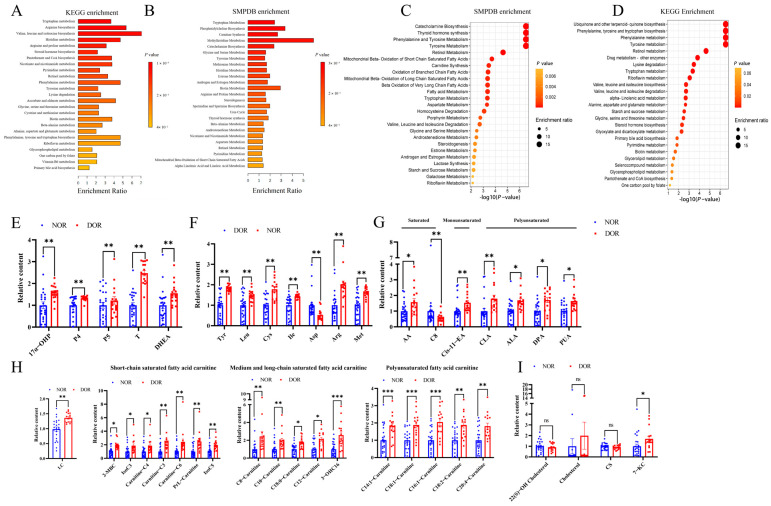
**Pseudo-targeted metabolomics analysis changes in human follicle fluid from NOR and DOR patients.** The follicle fluid collected from 25 NOR women and 16 DOR women was used for pseudo-targeted metabolomics analysis. MSEA was conducted using MetaboAnalyst 6.0 based on the KEGG database and SMPDB database using over-representation analysis (ORA) and quantitative enrichment analysis (QEA) methods. (**A**) KEGG enrichment analysis (ORA); (**B**) SMPDB enrichment analysis (ORA); (**C**) SMPDB enrichment (QEA); (**D**) KEGG enrichment (QEA). The levels of metabolites in steroid biosynthesis (**E**), amino acids (**F**), fatty acids (**G**), carnitines (**H**), and cholesterol-related metabolites (**I**). 17α-OHP: 17α-Hydroxyprogesterone, P4: Progesterone, P5: Pregnenolone, T: Testosterone, DHEA: Dehydroepiandrosterone, Tyr: L-Tyrosine, Leu: DL-Leucine, Cys: Cystine, Ile: Isoleucine, Asp: L-Aspartic acid, Arg: L-Arginine, Met: Methionine, AA: Azelaic acid, C8: Cap-rylic acid, Cis-11-EA: Cis-Gondoic acid, CLA: 10E,12Z-Octadecadienoic acid, ALA: Alpha-Linolenic acid, DPA: All-cis-4,7,10,13,16-docosapentaenoic acid, PUA: Punicic Acid, LC: L-Carnitine, 2-MBC: 2-Methylbutyroylcarnitine, IsoC3: Isobutyryl carnitine, C6-Carnitine: Hexanoylcarnitine, PrL-Carnitine: Propionyl-L-carnitin, IsoC5: Isovalerylcarnitine, C8-Carnitine: L-Octanoylcarnitine, C10-Carnit-ine: Decanoylcarnitin, C18: 0-Carnitine:Stearoylcarnitine, C12-Carnitine: Dodecanoy-lcarnitine, 3-OHC16: 3-Hydroxy-hexadecanoyl carnitine, C14: 1-Carnitine:Tetradecenylcarnitine, C18: 1-Carnitine: Oleoylcarnitin, C16: 1-Carniti-ne: Hexadecenoylcarnitine, C18: 2-Carnitine: Linoleoylcarnitine, C20: 4-Carnitine: Ar-achidonoylcarnitine, 22(S)-OH Cholesterol: 22(S)-Hydroxycholesterol, CS: Cholesteryl sulfate, 7-KC: 7-Ketocholes-terol. The data were expressed as mean ± SEM. ns: No Significant, * *p* < 0.05, ** *p* < 0.01, *** *p* < 0.001.

Pseudo-targeted metabolomic analysis has identified 903 metabolites in follicle fluid. Among them, 301 metabolites were increased, and 30 metabolites were decreased in DOR patients compared with healthy controls (Supplemental Figure S2B). Steroid hormone biosynthesis, tryptophan metabolism, lysine degradation, biotin metabolism, tyrosine metabolism, glycerophospholipid metabolism, valine, leucine, isoleucine biosynthesis, one-carbon pool by folate, etc., were enriched in KEGG using the ORA method (Figure 3A). Androgen and estrogen metabolism, tryptophan metabolism, steroidogenesis, estrone metabolism, histidine metabolism, carnitine synthesis, mitochondrial β-oxidation of short-chain saturated fatty acids, and phosphatidylcholine biosynthesis, etc., were enriched in SMPDB enrichment analysis using the ORA method (Figure 3B).

MSEA analysis using the QEA method showed that mitochondrial ß-oxidation of short chain saturated fatty acids, carnitine synthesis, oxidation of branched chain fatty acids, mitochondrial β oxidation of long-chain saturated fatty acids, β oxidation of very long chain fatty acids, androstenedione metabolism, steroidogenesis, estrone metabolism, and androgen and estrogen metabolism were enriched in the DOR group compared to the NOR group based on the SMPDB database (Figure 3C), and phenylalanine metabolism, phenylalanine, tyrosine, tryptophan biosynthesis, tyrosine metabolism, retinol metabolism, drug metabolism-other enzymes, lysine degradation, tryptophan metabolism, riboflavin metabolism, etc., were enriched in the DOR group based on the KEGG database (Figure 3D).

As shown in Figure 3E, the levels of steroid hormone metabolites, including 17α-hydroxyprogesterone, progesterone, pregnenolone, testosterone, and dehydroepiandrosterone, were elevated in the DOR group. Figure 3F showed that the levels of tyrosine, cystine, leucine, isoleucine, arginine, and methionine were increased, whilst aspartic acid level was decreased in the DOR group. Figure 3G displayed that the levels of some polyunsaturated fatty acids, monounsaturated fatty acid cis-gondoic acid, and saturated fatty acid azelaic acid were increased in DOR patients compared to NOR patients. L-carnitine levels were significantly elevated in the DOR group, accompanied by increases in short-chain acylcarnitines, medium- and long-chain acylcarnitines, and polyunsaturated acylcarnitines (Figure 3H). The levels of 7-ketocholesterol were increased, whereas levels of cholesteryl sulfate and cholesterol remained unchanged in the DOR group compared to the NOR group (Figure 3I). Additionally, various lysophospholipid levels were elevated in DOR patients, which included saturated lysophospholipids LysoPC 20:0, LysoPC 17:0, LysoPC 15:0, and LysoPC 14:0, monounsaturated lysophospholipids LysoPC 16:1 and LysoPC 15:1; and polyunsaturated lysophospholipids LysoPC 20:2, LysoPE 18:2, and LysoPC 16:2 [2N Isomer] (Supplementary Figure S4).

### 3.5. Apoptosis Rate Is Increased While Proliferative Rate Is Decreased and Ferroptosis Occurs in GCs of DOR Patients

**Figure 4:**
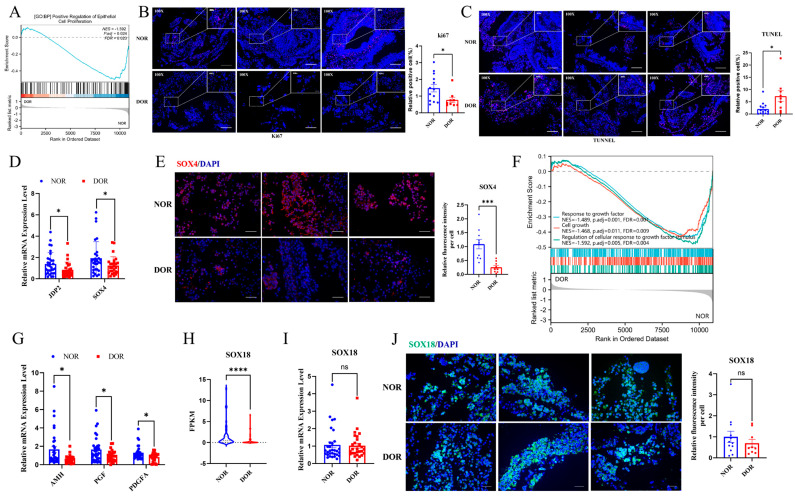
**Apoptosis rate is increased while proliferative rate is decreased in GCs of DOR patients.** GCs collected from 34 NOR women and 27 DOR women were used for Q-PCR validation, and GCs from 12 NOR women and 9 DOR women were fixed in 10% buffered formalin and subsequently used for IF staining. (**A**) GSEA analysis based on RNA-seq data. (**B**) IF staining of Ki67 (left) and the corresponding positive cell rate (right) (400×). (**C**) Tunel staining and the corresponding positive cell rate (right) (400×). (**D**) The mRNA levels of JDP2 and SOX4 were determined by Q-PCR. (**E**) Representative images of IF staining of SOX4 (left panel, 400×) and the cumulative diagram of relative fluorescence intensity/cell (right panel). (**F**) GSEA analysis based on RNA-seq data. (**G**) The mRNA levels of AMH, PGF, and PDGFA were determined by Q-PCR. (**H**) The FPKM values of SOX18 based on RNA-seq data. (**I**) The mRNA levels of SOX18 were determined by Q-PCR. (**J**) Representative images of IF staining of SOX18 (left, 400×) and the cumulative diagram of relative fluorescence intensity/cell (right). The data were expressed as mean ± SEM. ns: No Significant, * *p* < 0.05, *** *p* < 0.001, **** *p* < 0.0001.

GSEA showed that positive regulation of epithelial cell proliferation was downregulated in the DOR group (Figure 4A). We confirmed that Ki67 staining was significantly decreased whilst TUNEL staining was significantly increased in the DOR group (Figure 4B,C). The mRNA levels of JDP2, a transcriptional factor (TF) of repressing apoptosis, and SOX4, a TF related to GC proliferation and survival, were reduced in the DOR group compared to the NOR group (Figure 4D). SOX4 protein expression was robustly decreased in the DOR group compared to the NOR group (Figure 4E).

GSEA showed that cell growth, response to growth factors, and regulation of cellular response to growth factor stimulus were downregulated in the DOR group (Figure 4F). Figure 4G shows that the growth factors AMH, PGF, and PDGFA mRNA levels were significantly reduced in DOR patients compared to NOR patients (Figure 4G). Of note, SOX18, a TF related to the regulation of cell growth and response to growth factors, was a DEG in DOR vs. NOR (Figure 4H). SOX18 mRNA and protein levels were reduced in the DOR group without statistical significance (Figure 4I,J).

**Figure 5:**
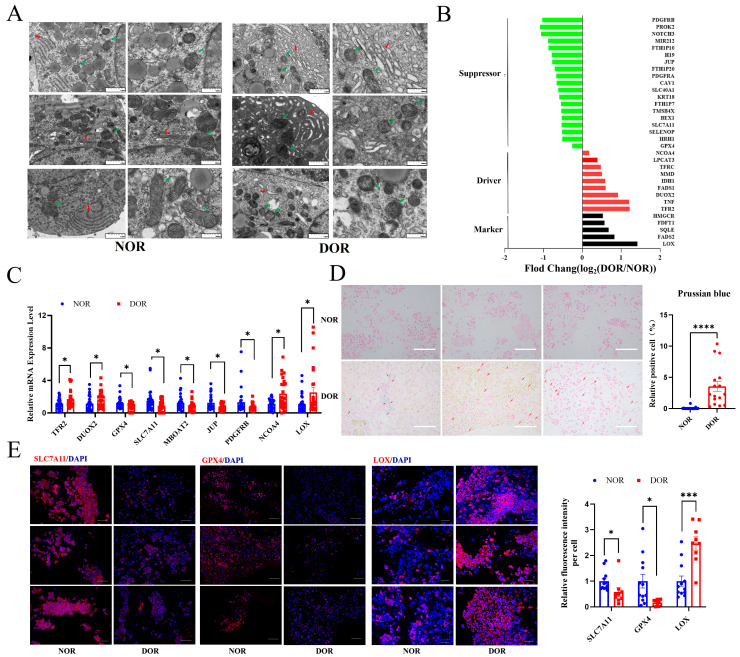
**Ferroptosis occurs in GCs of DOR patients.** GCs collected from 34 NOR women and 27 DOR women were used for Q-PCR validation, and GCs from 12 NOR women and 9 DOR women were used for IF. GCs from 7 NOR women and 7 DOR women were used for TEM analysis. (**A**) Representative TEM images of the GCs (left, 6.0k×; right, 12.0k×). The green arrowhead indicates the mitochondria, and the red arrowhead indicates the endoplasmic reticulum. (**B**) Changed genes associated with ferroptosis. (**C**) The mRNA levels of TFR2, DUOX2, GPX4, SLC7A11, MBOAT2, JUP, PDGFRB, NCOA4, and LOX were determined by Q-PCR. (**D**) Prussian blue staining of GCs. Light panel, representative images. The red arrowhead indicates the positive Prussian blue granular cells. Right panel: cumulative diagram of the rate of positive cells (200×). (**E**) IF staining of SLC7A11, GPX4, and LOX. Left panel: the representative images (400×). Right panel: cumulative diagram of relative fluorescence intensity/cell. The data were expressed as mean ± SEM. * *p* < 0.05, *** *p* < 0.001, **** *p* < 0.0001.

TEM displayed that the mitochondria underwent shrinkage in size, an increase in membrane density, and a reduction in or disappearance of mitochondrial cristae, indicating ferroptosis occurring in the DOR group (Figure 5A). The endoplasmic reticulum exhibited significant swelling in the DOR group.

We then analyzed the expression of the DEGs related to ferroptosis in DOR/NOR using the ferroptosis database FerrDb (http://www.zhounan.org/ferrdb/, accessed on 15 May 2024) [18] and KEGG database (https://www.kegg.jp/, accessed on 15 May 2024). Figure 5B showed the genes linked to ferroptosis, including SLC7A11, GPX4, FADS1, FADS2, LOX, IDH1, DUOX2, LPCAT3, SLC40A1, JUP, etc., in DEGs of DOR/NOR patients in transcriptome analysis. We then confirmed that TFR2 and DUOX2 mRNA levels were increased, whereas GPX4 and SLC7A11 levels were decreased in DOR patients (Figure 5C). In MBOAT2, JUP, and PDGFRB, the endogenous ferroptosis suppressor, mRNA levels were downregulated, while in NCOA4, a factor promoting ferroptosis, and lysyl oxidase (LOX), a novel contributor of ferroptosis, mRNA expression levels were upregulated in the DOR group compared to the NOR group (Figure 5C).

Positive Prussian blue staining was found in the DOR group. In contrast, there was no positive Prussian blue staining found in the NOR group (Figure 5D). SLC7A11 and GPX4 protein levels were decreased, whilst LOX staining was robustly increased in the DOR group (Figure 5E). Consistent with the SLC7A11 expression level, the level of cystine in follicle fluid was significantly increased in DOR patients compared to the NOR group (Figure 3F).

### 3.6. Reduced FSHR Signaling and Impaired Conversion of Androgen to Estrogen in GCs of DOR Patients

**Figure 6:**
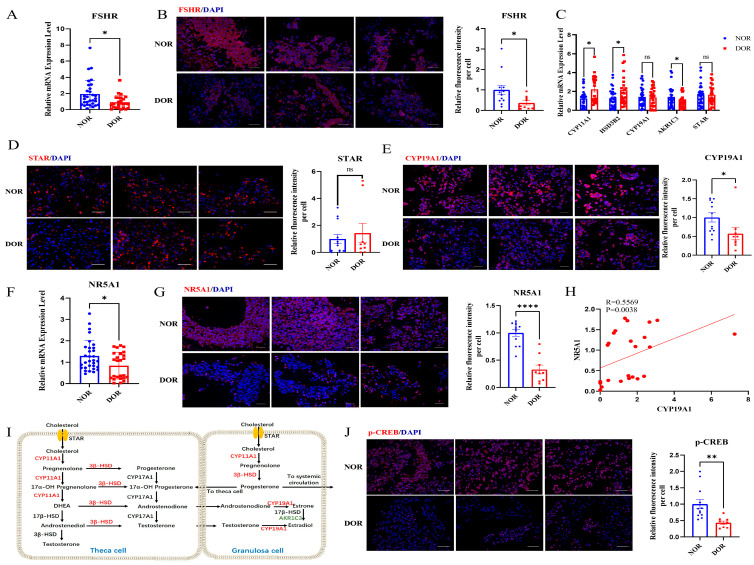
**Reduced FSHR signaling and changed expression of the key enzymes in steroid hormone in GCs of DOR patients.** GCs collected from 34 NOR women and 27 DOR women were used for Q-PCR validation, and GCs from 12 NOR women and 9 DOR women were used for IF staining. (**A**) The mRNA levels of FSHR were determined by Q-PCR. (**B**) Representative images of IF staining of FSHR (left, 400×) and the cumulative diagram of relative fluorescence intensity/cell (right). (**C**) The mRNA levels of CYP11A1, HSD3B2, CYP19A1, AKR1C3, and STAR were determined by Q-PCR. (**D**) Representative images of IF staining of STAR (left, 400×) and cumulative diagram of relative fluorescence intensity/cell (right). (**E**) Representative images of IF staining of CYP19A1 (left, 400×) and cumulative diagram of relative fluorescence intensity/cell (right). (**F**) The mRNA levels of NR5A1 were determined by Q-PCR. (**G**) Representative images of NR5A1IF staining (left, 400×) and the cumulative diagram of relative fluorescence intensity/cell (right). (**H**) The correlation between NR5A1 mRNA expression and CYP19A1 mRNA expression. (**I**) Schematic map of steroidogenesis in ovary. Red letters indicate regulation; green letters indicate downregulation. (**J**) Representative images of IF staining of p-CREB (left, 400×) and the cumulative diagram of relative fluorescence intensity/cell (right). The data are expressed as mean ± SEM. ns:No Significant, * *p* < 0.05, ** *p* < 0.01, **** *p* < 0.0001.

As expected, the FSHR mRNA level and FSHR-positive staining were substantially reduced in DOR patients compared to the NOR group (Figure 6A,B). As mentioned, MSEA showed that androstenedione metabolism, steroidogenesis, estrone metabolism, androgen, and estrogen metabolism were enriched in the follicle fluid of the DOR group (Figure 3D). We therefore examined the expression levels of the key genes associated with steroid hormone biosynthesis (Figure 6I). Q-PCR showed that CYP11A and HSD3B2 mRNA levels were significantly increased while AKR1C3 levels were significantly decreased in DOR patients compared to the NOR group (Figure 6C). However, STAR and CYP19A1 mRNA levels did not differ among DOR and NOR patients. IF showed that STAR protein expression did not differ between DOR and NOR groups (Figure 6D), but CYP19A1 protein expression was significantly reduced in the DOR group compared to the NOR group (Figure 6E).

NR5A1, also known as steroidogenic factor 1 (SF-1), is a master regulator of steroid hormone biosynthesis in granulosa cells by regulating transcription of CYP19A1, CYP11A1, HSD3B, STAR, etc. [19,20]. NR5A1 mRNA and protein expressions were significantly decreased in the DOR group compared to the NOR group (Figure 6F,G). Interestingly, the NR5A1 mRNA level was correlated with the CYP19A1 level (Figure 6H). Phosphorylated CREB-ser133, a key downstream factor of FSHR signaling, regulates CYP19A1 [21], CYP11A1 [22], and STAR [23] expression and suppresses apoptosis in GCs [24]. As shown in Figure 6J, phosphorylated CREB-ser133 expression was significantly reduced in the DOR group compared to the NOR group.

Given that the levels of progesterone and androgen were increased in the follicle fluid of the DOR group, we examined the receptors of steroid hormones in GCs. We note that estrogen receptors ESR1 and ESR2, androgen receptor AR, and progesterone receptor PR were not DEGs in DOR vs. NOR. We then confirmed that ESR1, ESR2, PR, and AR mRNA expression levels did not differ between DOR and NOR (Supplemental Figure S5).

### 3.7. Abnormal Cholesterol and Fatty Acid Synthesis in GCs from DOR Patients

**Figure 7:**
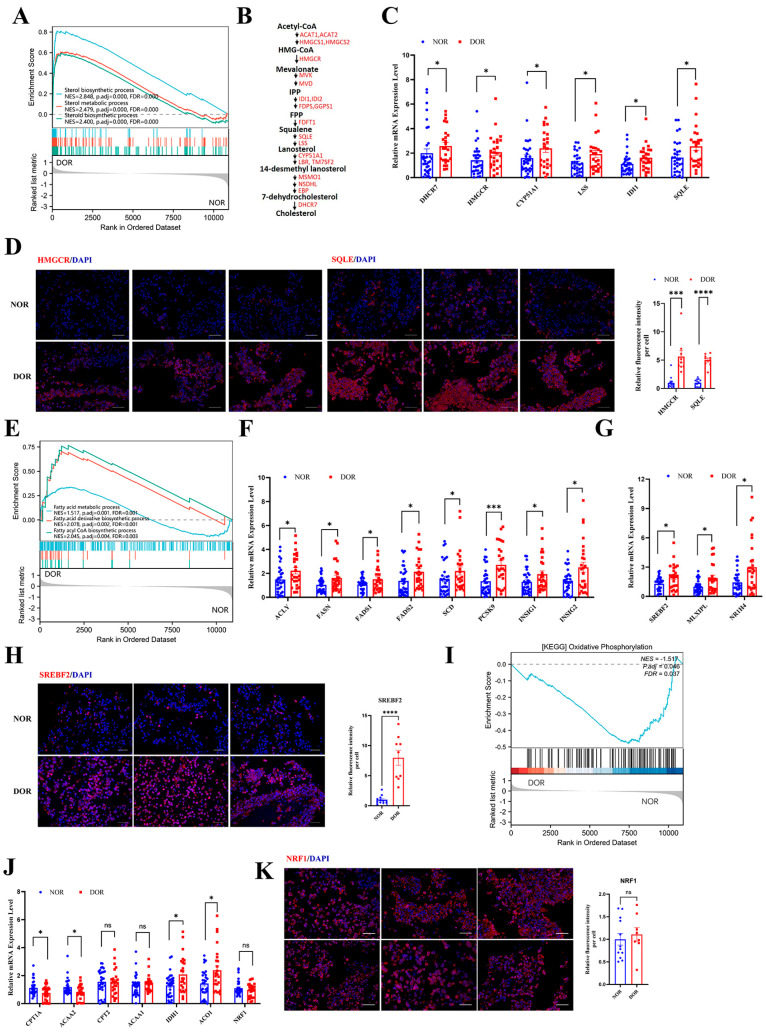
Abnormal cholesterol, fatty acid synthesis, fatty acid β oxidation, and TCA cycle are impaired in GCs from DOR patients. (**A**) GSEA analysis based on RNA-seq data. (**B**) Diagrammatic sketch map of cholesterol synthesis. (**C**) The mRNA levels of DHCR7, HMGCR, CYP51A1, LSS, IDI1, and SQLE were determined by Q-PCR. (**D**) Representative images of IF staining of HMGCR and SQLE (left, 400×) and the cumulative diagram of relative fluorescence intensity/cell (right). (**E**) GSEA analysis based on RNA-seq data. (**F**) The mRNA levels of ACLY, FASN, FADS1, FADS2, SCD, PCSK9, INSIG1, and INSIG2 were determined by Q-PCR. (**G**) The mRNA levels of SREBF2, MLXIPL, and NR1H4 were determined by Q-PCR. (**H**) Representative images of SREBF2 IF staining (400×) and the cumulative diagram of relative fluorescence intensity/cell. (**I**) GSEA analysis based on RNA-seq data. (**J**) The mRNA levels of CPT1A, ACAA2, CPT2, ACAA1, IDH1, ACO1, and NRF1 were determined by Q-PCR. (**K**) Representative images of NRF1 IF staining (400×) and the cumulative diagram of relative fluorescence intensity/cell. The data are expressed as mean ± SEM. ns: No Significant, * *p* < 0.05, *** *p* < 0.001, **** *p* < 0.0001.

As mentioned, GO enrichment indicated cholesterol synthesis enriched in the DOR group, and PPI showed some genes in cholesterol biosynthesis were the hub genes in DEGs of DOR vs. NOR (Figure 1). GSEA analysis showed that the sterol biosynthetic process, sterol metabolic process, and steroid biosynthetic process were upregulated in the DOR group (Figure 7A). It was found that many enzymes of cholesterol synthesis, such as ACAT2, HMGCR, MVD, IDI1, IDI2, CYP 51A1, FDPS, FDFT1, SQLE, LSS, DHCR7, etc., were the DEGs in transcriptome analysis (Figure 7B, Supplemental Figure S6). Q-PCR confirmed that DHCR7, HMGCR, CYP51A1, LSS, IDI1, and SQLE mRNA levels were significantly upregulated in the DOR group compared to the NOR group (Figure 7C). As shown in Figure 7D, HMGCR and SQLE protein expression were significantly increased in DOR patients compared to NOR patients.

GSEA showed that the pathways related to the fatty acid metabolic process, saturated fatty acid derivative biosynthetic process, and fatty acy-CoA biosynthetic process were upregulated in the DOR groups (Figure 7E). We then validated that the key genes linked to fatty acid synthesis ACLY and FASN, and unsaturated fatty acid biosynthesis process FADS1, FADS2, and SCD mRNA levels were significantly increased in DOR patients compared to NOR controls (Figure 7F). The genes associated with the regulation of fatty acid biosynthesis process PCSK9, INSIG1, and INSIG2 mRNA levels were also increased in the DOR group (Figure 7F). Interestingly, metabolomic analysis showed increased levels of many polyunsaturated fatty acids in follicle fluid of DOR patients (Figure 3G).

The mRNA level of SREBF2, the key TF in regulation of cholesterol and fatty acid synthesis, was significantly upregulated in the DOR group compared to the NOR group (Figure 7G). Other TFs associated with the fatty acid biosynthesis and metabolism process MLXIPL mRNA level and the TFs related to steroid biosynthesis and fatty acid homeostasis NR1H4 mRNA level were also significantly increased in DOR patients (Figure 7G). Figure 7H shows that SREBF2-positive staining was significantly increased in DOR patients compared to the NOR group.

### 3.8. Fatty Acid β Oxidation and TCA Cycle Are Impaired in DOR Group

GSEA KEGG showed downregulation of oxidative phosphorylation in the DOR group (Figure 7I), supporting mitochondrial dysfunction occurring in DOR patients. Of note, metabolomic analysis showed increased levels of various acylcarnitines in the follicle fluid of the DOR group (Figure 3H), suggesting that fatty acid β oxidation is impaired in the DOR group. Q-PCR confirmed that CPT1A and ACAA2 mRNA levels, but not CPT2 and ACAA1 mRNA levels, were reduced in the DOR group compared to the NOR group (Figure 7J). Of note, the mRNA levels of IDH1 and ACO1, the genes in the TCA cycle, were increased in DOR patients (Figure 7J). We noted that NRF-1, a critical TF in the regulation of mitochondrial biogenesis and fatty acid metabolism, was a DEG in DOR vs. NOR groups. We validated that the NRF-1 mRNA level and protein level did not differ between the DOR and NOR groups (Figure 7J,K).

Q-PCR showed that SLC2A6, PFKP, PGM1, LDHA, PFKFB4, and TKT levels were significantly increased in the DOR group compared to the NOR group (Supplemental Figure S7). HIF1α, a TF related to glycolysis and apoptosis, mRNA level, and protein level (Supplemental Figure S8) did not significantly differ between the DOR and NOR groups.

### 3.9. CYP19A1 Level Is Correlated with the Levels of Factors Linked to Ferroptosis, Whilst CYP11A1 and HSD Levels Are Correlated with the Level of Genes Related to Cholesterol Biosynthesis in DOR Patients

**Figure 8:**
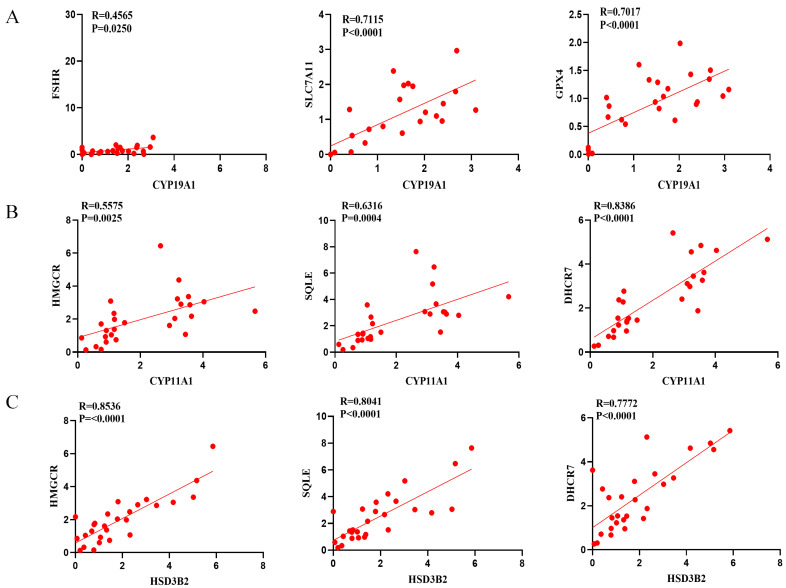
The correlations of CYP19A1 and FSHR levels with the levels of the genes linked to ferroptosis and cholesterol synthesis. (**A**) The correlations of CYP19A1 level with FSHR and CYP19A1 level with SLC7A11 and GPX4 levels. (**B**) The correlations of CYP11A1 level with HMGCR, SQLE, and DHCR7 levels. (**C**) The correlations of HSD3B2 level with HMGCR, SQLE, and DHCR7 levels.

We then analyzed the correlations between variables in the DOR group using Pearson’s correlation analysis. As shown in Figure 8A, the CYP19A1 mRNA level was positively correlated with the FSHR level. Moreover, the CYP19A1 level was positively correlated with the SLC7A11 and GPX4 levels. CYP11A1 and HSD3B2 levels were correlated with HMGCR, SQLE, and DHCR7 levels (Figure 8B,C).

## 4. Discussion

The present study has revealed the molecular networks associated with DOR disease in GCs using a relatively large sample size. Importantly, we have identified potential novel findings, including the occurrence of ferroptosis and the reprogramming of cholesterol and fatty acid synthesis and metabolism in GCs from DOR patients.

In this study, we have found that positive Prussian blue staining, decreased levels of SLC7A11 and GPX4, elevated LOX expression, and ultrastructural features of ferroptosis were observed in transmission electron microscopy images in GCs of DOR, which strongly indicates ferroptosis occurrence. Several studies have demonstrated increased apoptosis rate in GCs of DOR patients, which is associated with reduced AFCs and abnormal follicle development in these patients [25,26,27]. Our findings of ferroptosis occurrence in GCs indicate that several ways of cellular injury may exist in the GCs of DOR patients. Notably, we found that many genes linked to ferroptosis were enriched in DOR, such as transporters of Fe^2+^ TFR2 and SLC40A1, the enzymes related to lipid peroxidation LPCAT3, and ferritinophagy NCOA4. The ferroptosis suppressors of PDGFRB, JUP, and MBOAT2 mRNA levels were reduced in DOR patients. The oxidase gene DUOX2 mRNA expression was upregulated in the DOR group, suggesting oxidative stress that might contribute to ferroptosis.

Recent studies have suggested that enzymes in the mevalonate pathway may play critical roles in ferroptosis. For instance, DHCR7 acts as a pro-ferroptotic gene by converting 7-dehydrocholesterol (7-DHC) to cholesterol, as 7-DHC serves as an endogenous inhibitor of ferroptosis [28]. Similarly, SQLE is a crucial regulator of ferroptosis in various cells because it promotes carbon flux through squalene to cholesterol, thereby reducing CoQ10 and squalene, two novel lipid peroxide scavengers besides GPX4 [29,30]. Thus, SQLE has been implicated as a marker of ferroptosis [31]. Intriguingly, we observed significantly elevated mRNA levels of DHCR7 and SQLE, along with increased SQLE protein expression, in GCs from DOR patients. These data suggest that ferroptosis in GCs might be associated with dysregulation of these enzymes in DOR.

Several studies have reported associations between FSHR polymorphisms and conditions such as poor ovarian response [32] and primary ovarian insufficiency [33]. Consistent with these findings, we showed downregulated FSHR mRNA and protein expression in GCs from DOR patients. This reduction in FSHR expression may be attributed to decreased NR5A1 levels, as NR5A1 is a key transcription factor regulating FSHR expression in GCs [20]. FSHR activation would lead to phosphorylated CREB ser-133, thereby increasing CREB activity [34,35,36]. We found that phosphorylated CREB ser-133 expression level was significantly reduced in DOR patients, suggesting that FSHR signaling is decreased in GCs of these patients. It is well known that estrogen is mainly produced by GCs because these cells express aromatase (CYP19A1), a key enzyme converting androgen to estrogen [37]. We found protein levels of CYP19A1 were reduced in GCs from DOR patients, aligning with previous studies [8,38]. Furthermore, elevated levels of DHEA and testosterone in follicular fluid from DOR patients further support the notion of reduced CYP19A1 expression and activity in their GCs. Given that CYP19A1 expression is regulated by NR5A1 and CREB, its downregulation may be attributed to reduced NR5A1 levels and diminished CREB activity. Notably, we observed lower circulatory testosterone and estradiol (E2) levels on the day of HCG administration in DOR patients compared to those with normal ovarian reserve (NOR). The lower circulatory testosterone and E2 levels might be associated with reduced average AFCs in DOR patients.

Our data suggest that granulosa cells in DOR patients are exposed to a microenvironment characterized by elevated androgen levels during follicular development. Previous studies have shown that androgens can stimulate cholesterol synthesis and upregulate the expression of SREBF2 [39]. In line with this, our study found that androgen levels increased in the follicular fluid in the DOR group. This suggests that reduced CYP19A1 activity may lead to increased testosterone levels, thereby promoting enhanced cholesterol synthesis. Recent studies have further demonstrated that androgens can induce ferroptosis in granulosa cells [40] and Sertoli cells [41] by regulating endogenous ferroptosis suppressors, such as MBOAT1/2, which modulate sensitivity to iron-dependent phospholipid peroxidation. Consistent with these findings, we observed a positive correlation between CYP19A1 levels and the expression of SLC7A11 and GPX4, suggesting a potential link between ferroptosis and impaired androgen-to-estrogen conversion in GCs of DOR patients. Notably, the accumulation of androgens in mature follicles of DOR patients is likely due to reduced CYP19A1-mediated conversion of androgens to estrogens. While some studies have proposed dehydroepiandrosterone (DHEA) supplementation as a potential treatment for DOR patients to enhance steroidogenesis by providing precursors for estradiol and testosterone synthesis [42], further study is needed to evaluate its efficacy and clinical applicability.

GCs can synthesize C21 steroid progesterone (Δ4 pathways) using cholesterol as substrate [34]. In the present study, we observed significantly elevated expression of the Δ4 pathway enzymes, including CYP11A1 and HSD3B2, in DOR patients, although STAR expression remained unchanged compared to the NOR group. These findings were not consistent with a prior study reporting unchanged CYP11A1 expression in GCs of DOR patients [8]. Reduced expression of STAR and HSD3B in the young women with defective gonadotropin responsiveness has been reported by Bildik et al. [43]. Such a difference between the two studies might be attributed to the difference in sample size, 116 samples in our study vs. 40 samples in their study [43]. Moreover, we found that the metabolites progesterone and pregnenolone levels in the Δ4 pathways were increased in DOR patients, which supports the finding regarding increased expression of enzymes in the Δ4 pathways, although such changes, which were from thecal cells, could not be excluded. The potential mechanisms regarding increased Δ4 pathways remain unknown so far. However, the substrate availability of cholesterol may affect the synthesis of ovarian steroidogenesis [44]. Our findings of upregulated enzymes in the mevalonate pathway and correlation of CYP11A1 and HSD3B2 levels with the enzymes in the mevalonate pathway suggest that de novo cholesterol synthesis is sufficient to support steroidogenesis in DOR patients.

Our data that impaired fatty acid β-oxidation and a shift toward glycolysis, as well as abnormal morphology of mitochondria in the DOR group, indicate that mitochondrial dysfunction occurs in GCs of DOR. In fact, mitochondrial dysfunction has been reported in DOR GCs by some studies [45,46,47]. Additionally, we observed significantly elevated levels of 7-ketocholesterol, an oxidized cholesterol derivative, in the follicular fluid of DOR patients, possibly due to oxidative stress resulting from mitochondrial dysfunction. Notably, 7-ketocholesterol has been shown to induce lipid metabolic reprogramming and promote cholesterol ester accumulation in certain cell types [48], which may further exacerbate ferroptosis [49].

While ovarian reserve is known to decline with age, and aging is a recognized risk factor for DOR [2], few studies have explored molecular signaling pathways in GCs of DOR patients across different age groups. In the present study, we compared the molecular networks in GCs of DOR patients under and over 35 years old and identified the distinct signaling pathways in DOR ≥ 35 years old and DOR < 35 years old. The pathways related to cell differentiation and organ development were enriched in under 35 Y/O, whereas those linked to metabolism and leukocyte activation were enriched in the over 35 Y/O group. However, several pathways, including sterol biosynthesis, cholesterol biosynthesis, cholesterol metabolism, and steroid biosynthesis, were common to both age groups. Interestingly, the hub genes shared between the two groups were primarily involved in cholesterol biosynthesis and metabolism, suggesting that these processes represent core molecular pathways in DOR patients regardless of age.

There were several important limitations in the present study. For instance, (1) the data were from one center in the present study. Data from cross centers are required in the future study; (2) the reason and cause of the upregulated mevalonate pathway in GCs of DOR remains to be defined, although it might be associated with increased androgen levels. It requires to be intensively analyzed by using the clinical laboratory indexes; (3) the roles of ferroptosis in the pathogenesis of DOR remain to be validated by using intact animal studies.

In summary, the present study not only revealed reduced FSHR expression in GCs but also revealed the novel findings that abnormal sterol and cholesterol synthesis and metabolism, and ferroptosis occur in GCs of DOR patients. Reduced CYP19A1 expression and increased androgen levels in follicular fluid indicate impaired androgen-to-estrogen conversion in GCs in DOR patients. Given that androgen and an upregulated mevalonate pathway can induce ferroptosis, ferroptosis occurrence in GCs might be associated with impaired androgen-to-estrogen conversion and an increased upregulated mevalonate pathway in DOR. Our study highlights that the blockage of ferroptosis might be a potential therapeutic strategy for DOR.

## Figures and Tables

**Table 1:** Characteristics of patients with DOR and NOR.

	NOR (*n* = 107)	DOR (*n* = 105)	*p*-Value
Female age (y)	32.62 ± 3.96	35.68 ± 5.15	0.000
Bilateral basal AFC	15.40 ± 8.14	6.29± 2.91	0.000
BMI (kg/m^2^)	21.75 ± 2.68	22.13 ± 2.78	0.577
AMH (ng/mL)	3.71 ± 2.62	0.86 ± 0.38	0.000
Basal FSH (IU/L)	6.95 ± 2.36	9.17 ± 4.17	0.000
Basal E2 (pg/mL)	145.45 ± 108.17	156.96 ± 133.45	0.495
Basal LH (mIU/mL)	5.71 ± 3.13	4.74 ± 2.12	0.010
Basal P4 (ng/mL)	1.04 ± 0.55	1.29 ± 1.47	0.105
T (nmol/L)	1.79 ± 0.87	1.48 ± 0.68	0.009
Total FSH used (IU)	2118.45 ± 730.88	2797.30 ± 841.64	0.000
Days of Gn	9.68 ± 2.55	9.77 ± 2.16	0.768
FSH on the day of HCG (IU/L)	14.74 ± 4.52	19.71 ± 5.68	0.000
E2 on the day of HCG (pg/mL)	10,436.74 ± 6157.66	4837.75 ± 2391.99	0.000
LH on the day of HCG (mIU/mL)	3.33 ± 1.98	4.43 ± 2.60	0.001
P4 the day of HCG (ng/mL)	2.70 ± 1.35	1.97 ± 1.19	0.000

BMI, body mass index; AMH, anti-Müllerian hormone; FSH, follicle-stimulating hormone; LH, luteinizing hormone; E2, estrogen; P4, progesterone; T, testosterone; Gn, gonadotropins.

## Data Availability

Detailed description of methods and original data of this study are available from the authors upon reasonable request. RNA-seq data sets generated in this study have been deposited at the NCBI database (No. 24983398).

## Supplementary Materials

The following supporting information can be downloaded at: https://www.mdpi.com/article/10.3390/antiox14060749/s1, Table S1: List of Primers for qPCR. Table S2: List of Antibodies. Figure S1: Positive FSHR staining in GCs. Figure S2: Volcano plots of transcriptomic and metabolomic analyses. Figure S3: Overview of transcriptome analysis of patients under and over 35 years old. Figure S4: The levels of lysophospholipids in follicle fluids detected by Quasi-targeted metabolomics. Figure S5: The expression levels of ESR1, ESR2, PR and AR in GCs of NOR and DOR patients. Figure S6: The expression levels of the genes in cholesterol biosynthesis using RNA-seq. Figure S7: The expression levels of the genes in glycolysis pathway in GCs of NOR and DOR patients. Figure S8: mRNA and protein expression of HIF-1α in GCs of NOR and DOR patients.

## Abbreviations

The following abbreviations are used in this manuscript:

DOR	diminished ovarian reserve
NOR	normal ovarian reserve
GCs	granulosa cells
ART	assisted reproductive technology
IVF	in vitro fertilization
ICSI	intracytoplasmic sperm injection
AFC	antral follicle count
AMH	anti-Müllerian hormone
FSH	follicle-stimulating hormone
GnRH	gonadotropin-releasing hormone
HCG	human chorionic gonadotropin
LH	luteinizing hormone
E_2_	estradiol
P_4_	progesterone
T	testosterone
PPI	protein–protein interaction
IF	immunofluorescence (IF)
TEM	transmission electron microscopy
