# Supplementary material for: Ferroptosis and Sterol Biosynthesis Dysregulation in Granulosa Cells of Patients with Diminished Ovarian Reserve
Source: Antioxidants (Basel). 2025 Jun 17;14(6):749. doi: 10.3390/antiox14060749 (PMC12189052; doi:10.3390/antiox14060749)
Supplement: Supplementary file 1 [file antioxidants-14-00749-s001.zip › Supplementary materials2025.06.24.pdf]

Supplemental materials

Table S1. List of Primers for qPCR

Gene ID	Gene	Forward primer	Reverse primer
ENSG00000106327	TFR2	GCACCTCAAAGCCGTAGTGTAC	CCACCTGTTCATAGAGAGTCTGC
ENSG00000167468	GPX4	ACAAGAACGGCTGCGTGGTGAA	GCCACACACTTGTGGAGCTAGA
ENSG00000250033	SLC7A11	TCCTGCTTTGGCTCCATGAACG	AGAGGAGTGTGCTTGCGGACAT
ENSG00000143797	MBOAT2	GGGTATGACGAAAATGGAGCAGC	CTTTTGAGCCAAAGAGCTGTCTG
ENSG00000266412	NCOA4	GCTTGCTATTGGTGGAGTTCTCC	GCCATACCTCACGGCTTCTAAG
ENSG00000124766	SOX4	GACATGCACAACGCCGAGATCT	GTAGTCAGCCATGTGCTTGAGG
ENSG00000186187	NRF1	GGCAACAGTAGCCACATTGGCT	GTCGTCTGGATGGTCATCTCAC
ENSG00000147465	STAR	TACGTGGCTACTCAGCATCGAC	TCAACACCTGGCTTCAGAGGCA
ENSG00000140459	CYP11A1	TGGCATCCTCTACAGACTCCTG	CTTCAGGTTGCGTGCCATCTCA
ENSG00000203859	HSD3B2	CGCCTGTATCATTGATGTCTTTGG	CTGGTGTAGATGAAGACTGGCAC
ENSG00000137869	CYP19A1	GACGCAGGATTTCCACAGAAGAG	ATGGTGTCAGGAGCTGCGATCA
ENSG00000196139	AKR1C3	CCGAAGCAAGATTGCAGATGGC	GTGAGTTTTCCAAGGCTGGTCG
ENSG00000170820	FSHR	GGTTTGTCCTCACCAAGCTTCG	GGTTGGAGAACACATCTGCCTC
ENSG00000140044	JDP2	CACTTCCTGGAGGTGAAACTGG	GAAACTCCGTGCGCTCCTTCTT
ENSG00000136931	NR5A1	CCAGACCTTCATCTCCATCGTG	TGGCGGTAGATGTGGTCGAACA
ENSG00000172893	DHCR7	TCCACAGCCATGTGACCAATGC	CGAAGTGGTCATGGCAGATGTC
ENSG00000116133	DHCR24	CAGGAGAACCACTTCGTGGAAG	CCACATGCTTAAAGAACCACGGC
ENSG00000113161	HMGCR	GACGTGAACCTATGCTGGTCAG	GGTATCTGTTTCAGCCACTAAGG
ENSG00000160285	LSS	GACGACCGATTCACCAAGAGCA	AGACATGCTCCTGGAAGGCAGT
ENSG00000001630	CYP51A1	CTCTTACCAGGTTGGCTGCCTT	CTTGAGACTGTCTGCGTTTCTGG
ENSG00000067064	IDI1	GCCGCAGACTGTGCTCAAAGC	CCTGTTGCTTGTCGAGGTGGTT
ENSG00000104549	SQLE	CTCCAAGTTCAGGAAAAGCCTGG	GAGAACTGGACTCGGGTTAGCT
ENSG00000186480	INSIG1	TTTTCTCAGGAGGCGTCACGGT	TCCTTGCTCTCAGAATCGGTGG
ENSG00000125629	INSIG2	GTCAGTAGGACTGTGGTGGACT	GGCAACCAAGAACGGACATAGAG
ENSG00000140279	DUOX2	CAATGGCTACCTGTCCTTCCGA	GTCCTTGGAGAGGAAGCCATTC
ENSG00000169710	FASN	TTCTACGGCTCCACGCTCTTCC	GAAGAGTCTTCGTCAGCCAGGA
ENSG00000149485	FADS1	CTGTCGGTCTTCAGCACCTCAA	CTGGGTCTTTGCGGAAGCAGTT
ENSG00000099194	SCD	CCTGGTTTCACTTGGAGCTGTG	TGTGGTGAAGTTGATGTGCCAGC
ENSG00000134824	FADS2	TGCAACGTGGAGCAGTCCTTCT	GGCACATAGAGACTTCACCAGC
ENSG00000131473	ACLY	GCTCTGCCTATGACAGCACCAT	GTCCGATGATGGTCACTCCCTT
ENSG00000198911	SREBF2	CTCCATTGACTCTGAGCCAGGA	GAATCCGTGAGCGGTCTACCAT
ENSG00000009950	MLXIPL	GCGTTTTGACCAGATGCGAGAC	CGTTGAAGGACTCAAACAGAGGC
ENSG00000012504	NR1H4	ACTTCCGTCTGGGCATTCTGAC	GCTGTAAGCAGAGCATACTCCTC
ENSG00000067057	PFKP	AGGCAGTCATCGCCTTGCTAGA	ATCGCCTTCTGCACATCCTGAG

ENSG00000079739	PGM1	TGATGGACGCGAGCAAACTGTC	ATGTCCTCCACACTCTGCTTGC
ENSG00000134333	LDHA	GGATCTCCAACATGGCAGCCTT	AGACGGCTTTCTCCCTCTTGCT
ENSG00000114268	PFKFB4	GATCCTGAGGTCATAGCTGCCA	CTATCCAGGTCCTCATCTAGCG
ENSG00000163931	TKT	CCAAGTGATGGCGTTGCTACAG	TTGTCCGACCTGGAAGTCCTCA
ENSG00000138413	IDH1	CTATGATGGTGACGTGCAGTCG	CCTCTGCTTCTACTGTCTTGCC
ENSG00000122729	ACO1	TCCTCAGGTGATTGGCTACAGG	TCGGTCAGCAATGGACAACTGG
ENSG00000160326	SLC2A6	TCACCAAGTCCTTCCTGCCAGT	CACAGCAGCCTGTGAACACCAG
ENSG00000110090	CPT1A	GATCCTGGACAATACCTCGGAG	CTCCACAGCATCAAGAGACTGC
ENSG00000167315	ACAA2	GGCACTGAAGAAAGCAGGACTG	GTGACCCAAAGCAATGGCTCCT
ENSG00000113083	LOX	GATACGGCACTGGCTACTTCCA	GCCAGACAGTTTTCCTCCGCC
ENSG00000119630	PGF	GGCGATGAGAATCTGCACTGTG	ATTCGCAGCGAACGTGCTGAGA
ENSG00000104899	AMH	CGCTGCTTCACACGGATGACC	GGTGGCGACTCCTCGAGTTCC
ENSG00000197461	PDGFA	CAGCGACTCCTGGAGATAGACT	CGATGCTTCTCTTCCTCCGAATG
ENSG00000113721	PDGFRB	TGCAGACATCGAGTCCTCCAAC	GCTTAGCACTGGAGACTCGTTG
ENSG00000173801	JUP	ACCAGCATCCTGCACAACCTCT	GGTGATGGCATAGAACAGGACC
ENSG00000203883	SOX18	ACGCCTTCATGGTGTGGGCAAA	GTTCAGCTCCTTCCACGCTTTG
ENSG00000213857	β-Actin	CACCATTGGCAATGAGCGGTTC	AGGTCTTTGCGGATGTCCACGT
ENSG00000091831	ESR1	GCTTACTGACCAACCTGGCAGA	GGATCTCTAGCCAGGCACATTC
ENSG00000140009	ESR2	ATGGAGTCTGGTCGTGTGAAGG	TAACACTTCCGAAGTCGGCAGG
ENSG00000082175	PR	GTCGCCTTAGAAAGTGCTGTCAG	GCTTGGCTTTCATTTGGAACGCC
ENSG00000169083	AR	ATGGTGAGCAGAGTGCCCTATC	ATGGTCCCTGGCAGTCTCCAAA
ENSG00000169174	PCSK9	GACACCAGCATACAGAGTGACC	GTGCCATGACTGTCACACTTGC
ENSG00000157184	CPT2	GCAGATGATGGTTGAGTGCTCC	AGATGCCGCAGAGCAAACAAGTG
ENSG00000060971	ACAA1	GACAGGTCATCACGCTGCTCAA	CCAGGGTATTCAAAGACGGCAG
ENSG00000100644	HIF-1α	TATGAGCCAGAAGAACTTTTAGGC	CACCTCTTTTGGCAAGCATCCTG

Table S2. List of Antibodies

Antibody	Brand	Category Number
Phospho-CREB1(Ser133)	Proteintech	28792-1-AP
CYP19A1	Proteintech	16554-1-AP
FSH Receptor	RayBio	188-11092-1
HIF-1 alpha	Abcam	ab51608
HMGCR	Proteintech	13533-1-AP
KI67	Abcam	Ab1667
LOX (F-8)	Santa Cruz	sc373995
N-cadherin	Proteintech	22018-1-AP
NR5A1	Proteintech	18658-1-AP
NRF1/nuclear respiratory factor 1	Proteintech	83092-1-RR
SLC7A11/xCT	Proteintech	2684-1-AP
SOX4	Bioss	BS-11208R
SOX18	Santa Cruz	SC-166025
SQLE	Proteintech	12544-1-AP
SREBF2	Cusabio	CSB-PA666241LA01HU
Glutathione Peroxidase 4	Proteintech	67763-1-lg
STAR	Proteintech	80751-1-RR

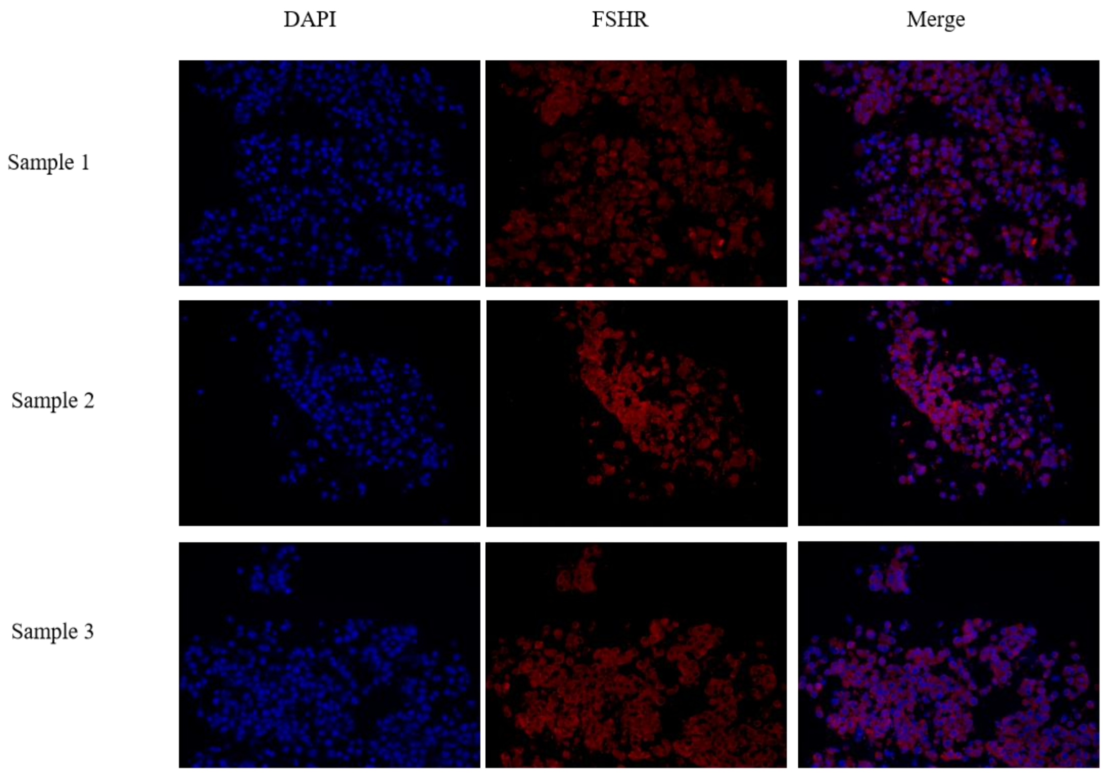

Figure S1. Positive FSHR staining in GCs. GCs were collected from patients who underwent ART. Representative images of FSHR staining showed that >97% of GCs were positive.

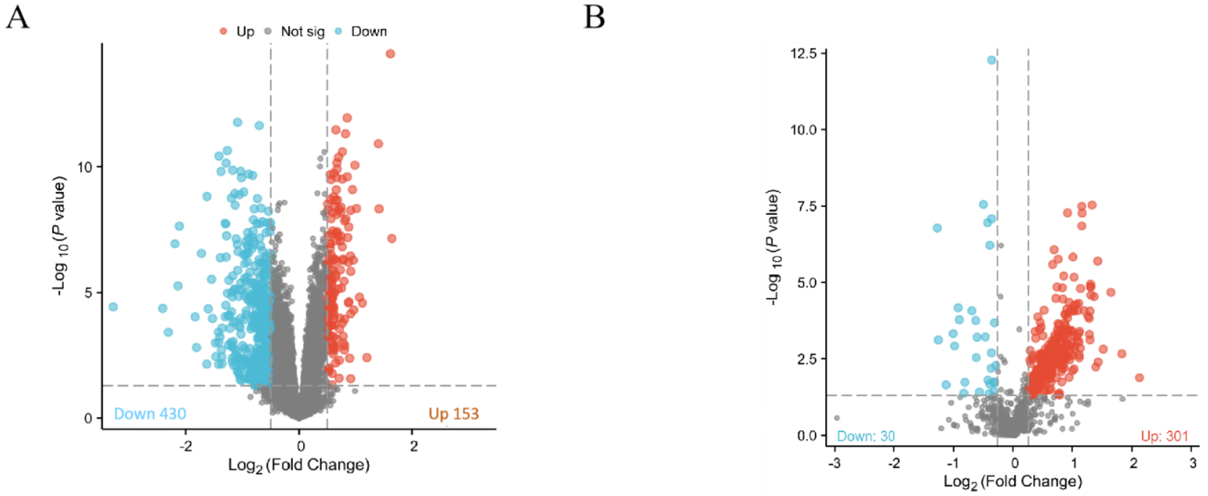

Figure S2. Volcano plots of transcriptomic and metabolomic analyses. GCs collected from 116 patients (62 DOR and 54 NOR) were used for RNA-seq, and follicle fluids were collected from 25 NOR and 16 DOR patients were used to detect metabolomics profile. (A) Transcriptome analysis revealed 583 differentially expressed genes (153 upregulated, 430 downregulated) in DOR versus NOR. （B） Pseudo-targeted metabolomic analysis identified alterations 331 metabolites between NOR and DOR, including 30 reduced metabolites and 301 increased metabolites.

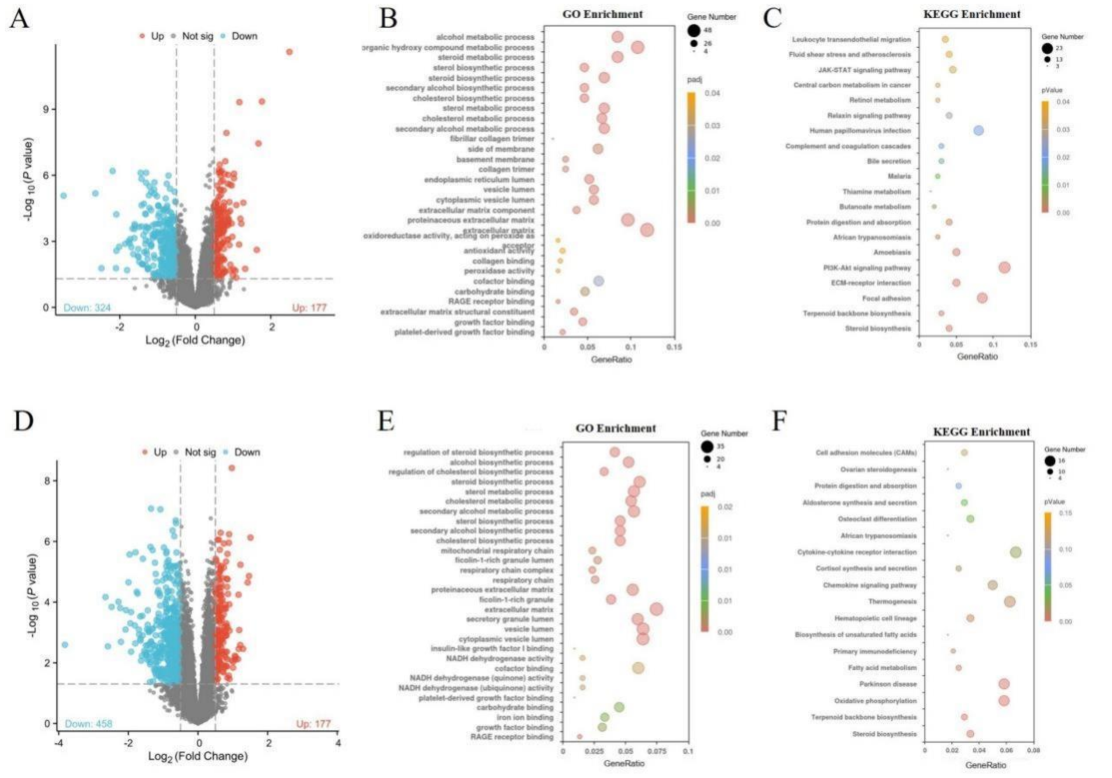

Figure S3. Overview of transcriptome analysis of patients under and over 35 years old. (A) In patients under 35 years old, 177 genes were upregulated and 324 genes were downregulated in DOR group compared to the age-matched NOR group. (B) GO enrichment analysis and (C) KEGG enrichment analysis of differential genes between NOR and DOR group under 35 years old. (D) In patients over 35 years old, 177 genes were upregulated and 458 genes were downregulated in DOR patients compared to the age-matched healthy group. (E) GO enrichment analysis and (F) KEGG enrichment analysis of differential genes between NOR and DOR group over 35 years old.

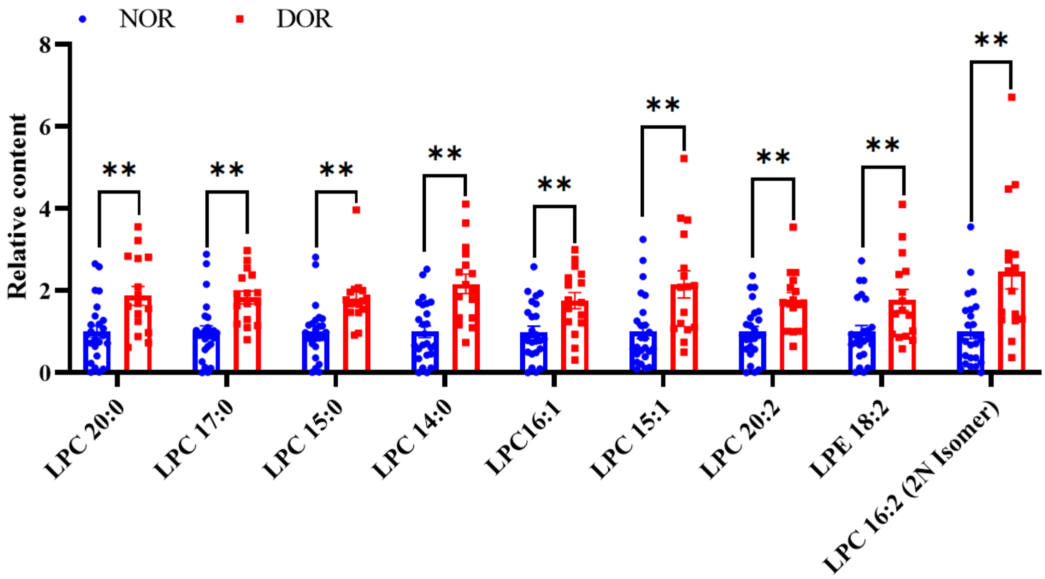

Figure S4. The levels of lysophospholipids in follicle fluids detected by Quasi-targeted metabolomics. The data were expressed as mean ± SEM. **p < 0.01.

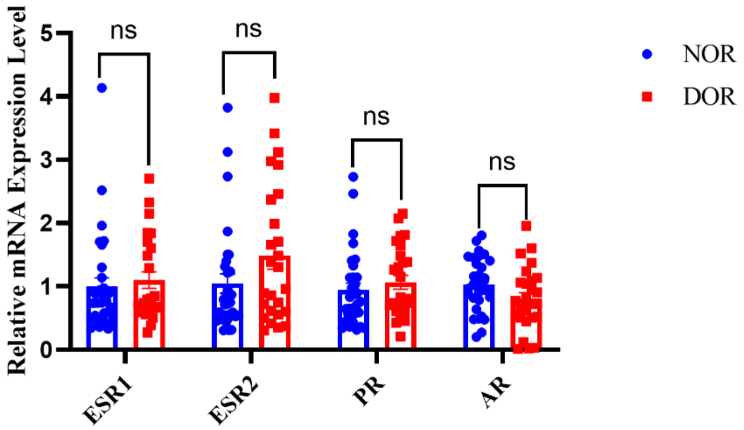

Figure S5. The expression levels of ESR1,ESR2,PR and AR in GCs of NOR and DOR patients. GCs collected from 34 NOR women and 27 DOR women were used for qPCR. The mRNA levels of ESR1, ESR2, PR and AR were determined by qPCR. ns:no significance.

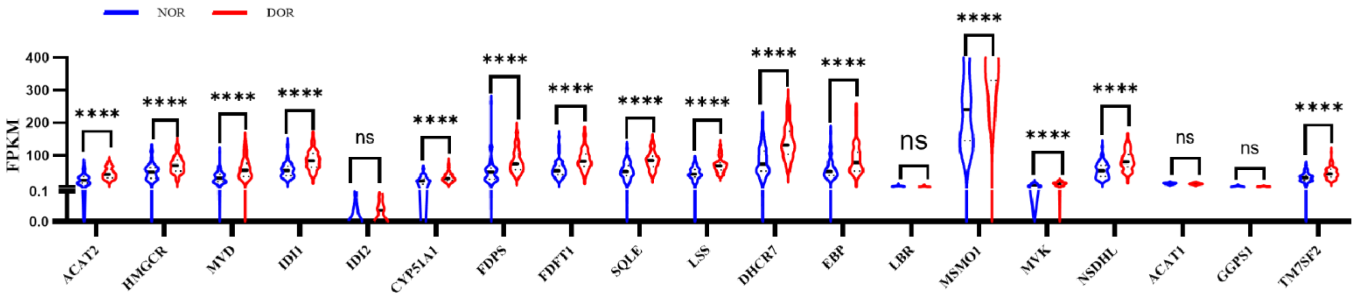

Figure S6. The expression levels of the genes in cholesterol biosynthesis using RNA-seq. GCs collected from 116 patients (62 DOR and 54 NOR) were used for RNA-seq. The FPKM values of ACAT2 , HMGCR, MVD, IDI1, IDI2, CYP51A1, FDPS, FDFT1, SQLE, LSS, DHCR7, EBP, LBR, MSMO1, MVK, NSDHL, ACAT1, GGPS1 and TM7SF2 based on RNA-seq data of NOR and DOR. The data were expressed as mean ± SEM. ****p < 0.0001, ns:no significance.

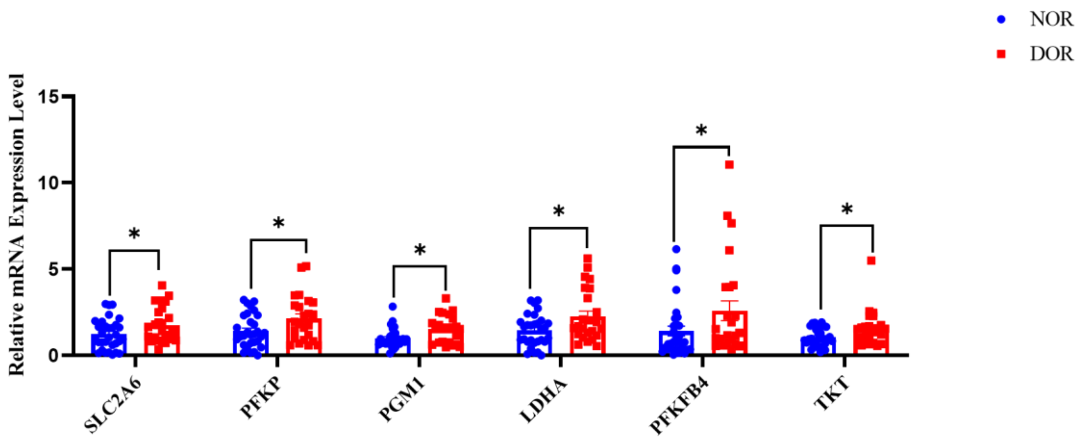

Figure S7. The expression levels of the genes in glycolysis pathway in GCs of NO R and DOR patients. The mRNA levels of SLC2A6 , PFKP, PGM1, LDHA, PFKFB4 and TKT were determined by qPCR. The data were expressed as mean ± SEM.*p < 0.05.

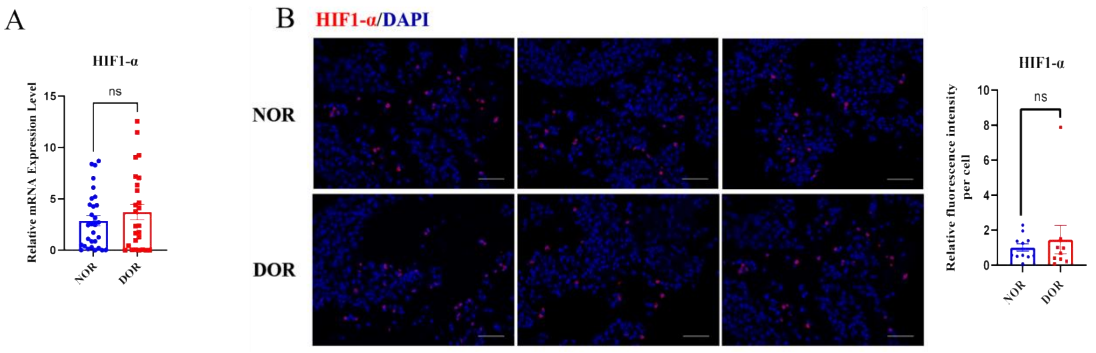

Figure S8. mRNA and protein expression of HIF-1α in GCs of NOR and DOR patients. (A) The mRNA levels of HIF-1 α were determined by qPCR. (B) Representative images of IF staining of HIF-1 α (left, 400×) and the cumulative diagram of relative fluorescence intensity /cell (right). The data were expressed as mean ± SEM. ns:no significance.
